# Impact of roxadustat on anemia management in infected patients undergoing long-term dialysis: a retrospective cohort analysis

**DOI:** 10.3389/fphar.2025.1695376

**Published:** 2025-10-08

**Authors:** Lulu Wang, Huimin Qiu, Xinqi Tan, Jing Liu, Ting Yang, Chunming Jiang

**Affiliations:** ^1^ Department of Pharmacy, Nanjing Drum Tower Hospital, Affiliated Hospital of Medical School, Nanjing University, Nanjing, China; ^2^ Hunan Provincial Key Laboratory of the Research and Development of Novel Pharmaceutical Preparations, the “Double-First Class” Application Characteristic Discipline of Hunan Province (Pharmaceutical Science), Changsha Medical University, Changsha, China; ^3^ Department of Nephrology, Nanjing Drum Tower Hospital Clinical College of Traditional Chinese and Western Medicine, Nanjing University of Chinese Medicine, Nanjing, China; ^4^ Department of Pharmacy, Nanjing Drum Tower Hospital, School of Basic Medicine and Clinical Pharmacy, China Pharmaceutical University, Nanjing, China; ^5^ Department of Nephrology, Nanjing Drum Tower Hospital, Nanjing, China

**Keywords:** renal anemia, roxadustat, chronic kidney disease, peritoneal dialysis, overt infection

## Abstract

**Introduction:**

The efficacy of oral roxadustat in dialysis patients with renal anemia and overt infections remains uncertain.

**Objectives:**

In this retrospective cohort study, 2816 such patients were screened, of whom 167 were enrolled and assigned to either roxadustat (n = 88) or recombinant human EPO (rHuEPO; n = 79) for treatment.

**Methods and results:**

Baseline hemoglobin levels were 90.3 ± 15.2 g/L and 91.9 ± 17.8 g/L, respectively. All patients received a mean of 10.6 ± 3.3 days of infection treatment. Types of infection included pulmonary, peritoneal dialysis-associated peritonitis, catheterrelated, urinary tract, and others. Compared with rHuEPO, roxadustat led to significantly greater increases in hemoglobin and ΔHb, and more pronounced improvements in ferritin, transferrin saturation (TSAT), and hepcidin levels following infection. The analysis demonstrated that TSAT, hepcidin, dialysis modality, residual renal function, infection type, and PCT levels play critical roles in mediating the efficacy of roxadustat under active infection conditions. Among peritoneal dialysis patients, roxadustat was associated with significantly greater improvements in ΔHb, ferritin, and TSAT compared to rHuEPO. ΔHb values varied by infection type, with significantly higher increases in peritoneal dialysis-associated peritonitis and a trend toward larger ΔHb in other infections relative to those in pulmonary infections. Based on procalcitonin levels, more severe infections were correlated with lower ΔHb values.

**Conclusion:**

Overal, roxadustat was more effective than rHuEPO in ameliorating renal anemia in infected dialysis patients.

## Introduction

Renal anemia represents a common and consequential complication in patients with chronic kidney disease (CKD), markedly diminishing quality of life and adversely affecting long-term prognosis (Zhu et al., 2017). Conventional treatment primarily involves recombinant human erythropoietin (rHuEPO); however, a substantial number of patients exhibit hyporesponsiveness, frequently associated with systemic inflammation and functional iron deficiency.

Roxadustat, a first-in-class oral hypoxia-inducible factor prolyl hydroxylase inhibitor (HIF-PHI), has emerged as a novel therapeutic alternative by stimulating endogenous erythropoietin synthesis and enhancing iron metabolism (Dhillon, 2019). Phase III clinical trials have demonstrated that roxadustat effectively elevates hemoglobin levels even in the presence of microinflammation, suggesting a potentially distinct advantage in inflammatory conditions compared to rHuEPO (Akizawa et al., 2020).

Nevertheless, the efficacy and hematopoietic response of roxadustat in the context of overt infection (a frequent and serious comorbidity in dialysis populations) remain insufficiently elucidated. Such infections can exacerbate anemia through pronounced inflammatory pathways, yet robust clinical evidence supporting roxadustat’s use in this setting is lacking (Weiss et al., 2019). In addition, factors such as age and gender, residual renal function, iron deficiency, hyperphosphatemia, deficiency of folate or vitamin B12, hyper-parathyroidism, use of ACEIs or ARBs, and the etiology of end-stage renal disease may also confound the clinical efficacy of roxadustat or human recombinant erythropoietin in dialysis patients (Wu et al., 2024; Fernández-Gallego et al., 2000). Therefore, this study aimed to retrospectively evaluate clinical data from dialysis patients with overt infections at our institution, with the objective of assessing the therapeutic effects of roxadustat on anemia correction and hematopoiesis-related outcomes in this clinically complex scenario.

## Methods

### General information

This was a retrospective study that reviewed the clinical data of patients with renal anemia who were treated with roxadustat or rHuEPO at Nanjing Drum Tower Hospital from January 1, 2020, to June 10, 2022. This study was approved by the Medical Ethics Committee of Nanjing Drum Tower Hospital (Ethics Code: 2023-118-01). All methods were carried out in accordance with relevant guidelines and regulations. Based on ethical approval, the data were accessed for research purposes from March 2023 to June 2024. Jing Liu and Chunming Jiang were able to identify individual participants during or after data collection.

The inclusion criteria were as follows: (1) aged 18 years or older, (2) duration of maintenance dialysis >3 months, (3) received roxadustat or rHuEPO treatment for at least 4 consecutive weeks, and (4) evidence of overt clinical infection. This evidence included 1) peritoneal dialysis-associated peritonitis meeting the ISPD guidelines (Li et al., 2022), 2) pulmonary infection, which was defined as respiratory infection with evidence of radiographic and/or hematologic pathogens (Carmona et al., 2024), and 3) other infections, which were defined as urinary tract infections and catheter-related infections diagnosed according to relevant guidelines (The General Office of the National Hea lth Commission, 2021; Takahashi et al., 2021; Emergency Medicine Branch of Chinese Medical Doctor Association et al., 2020). After screening, a total of 167 patients were eventually enrolled and divided into a roxadustat group and an rHuEPO group according to medication.

### Observed indicators

To explore the effect of roxadustat or rHuEPO on dialysis patients with renal anemia during overt infection, we analyzed data at 3 time points: (a) Time to overt infection (T1) was defined as the time of the first examination on hospital admission after the onset of symptoms of overt infection. (b) The time (T2) for the index of patient improvement was defined as the time of the last examination of improvement in infection symptoms before hospital discharge. (c) T3 was the follow-up time point approximately 1 month after discharge.

Primary indicators included the following: (1) anemia-related factors, such as hemoglobin (Hb), reticulocytes, folate, vitamin B12, serum ferritin, iron metabolism parameters, and red cell volume width (%); (2) type of infection, including pulmonary infection, peritoneal dialysis-related infection, catheter-related infection, and urinary tract infection; and (3) infection-related indicators, including CRP, WBC, and neutrophil levels. Other factors included baseline patient demographics, hemodialysis (HD), peritoneal dialysis (PD) and dialysis age, residual renal function, hyperphosphatemia, hyperparathyroidism, use of ACEIs or ARBs, and the etiology of end-stage renal disease.

### Therapeutic regimen

For the management of renal anemia, both roxadustat (oral administration) and rHuEPO (subcutaneous injection) were administered in accordance with the prescribing information and Kidney Disease: Improving Global Outcomes (KDIGO) guidelines. Iron therapy, either oral or intravenous, was provided based on individual patient requirements and prevailing treatment guidelines. For the treatment of patients with active infections, we strictly adhered to internationally recognized guidelines corresponding for the specific type of infection, and antibiotics were selected based on etiological evidence, in line with guideline recommendations. All treatment regimens were determined by a nephrologist.

### Assessment of iron metabolism and hepcidin levels

Ferritin, serum iron, total iron-binding capacity (TIBC), and transferrin saturation were extracted from electronic medical records. Serum hepcidin levels were quantified using a commercially available enzyme-linked immunosorbent assay (ELISA) kit (ZCIBIO Technology Co., Ltd., Shanghai, China), following the manufacturer’s protocols.

### Statistical methods

Statistical data were analyzed using SPSS 26.0 statistical software, with counting data expressed as [cases (%)] using the χ2 test and with counting data expressed as (x ± s) using the t-test for comparisons between groups and multi-time-point comparisons using one-way analysis of variance, with *P* < 0.05 indicating statistical significance. Missing observations were excluded from the analysis. Multiple logistic regression was used to identify factors associated with clinical efficacy. The 95% confidence intervals for treatment differences were based on the least squares method. To demonstrate noninferiority between roxadustat and rHuEPO, the lower 95% confidence interval for variation in hemoglobin levels was greater than or equal to −1.0 g/dL.

## Results

### General baseline data

**FIGURE 1:**
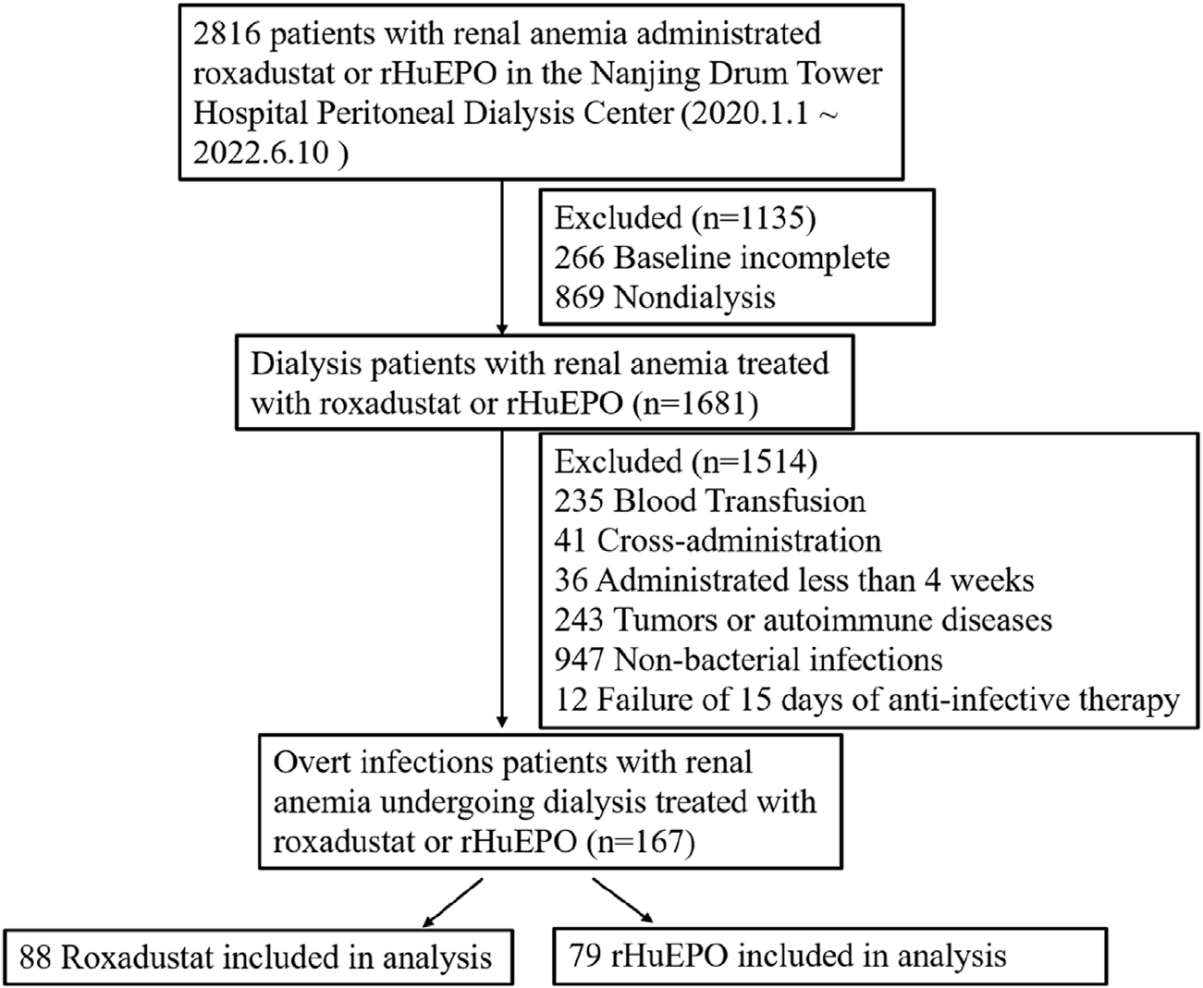
Enrollment screening process.

A total of 2816 patients with renal anemia treated with roxadustat or rHuEPO, and 167 dialysis patients with renal anemia and overt infections were ultimately assessed according to the inclusion and exclusion criteria and the diagnosis of overt infections; the patients included 88 patients treated with roxadustat and 79 patients treated with rHuEPO (Figure 1).

Of the 167 patients enrolled, 73 (43.7%) were on hemodialysis (HD) and 94 (56.3%) on peritoneal dialysis (PD). The overall baseline mean hemoglobin level was 9.2 g/dL. The baseline transferrin saturation in both groups were 28.7 (18.2, 35.8)%, and 18.9 (14.3, 33.2)%, respectively. The iron supplements and iron-based phosphate binders were not administered between both groups during the infection. Elevated C-reactive protein levels (>9.0 mg/L) were observed in 85% (n = 148) of the patients, while 81.4% (n = 136) had procalcitonin levels above the upper limit of normal. After adjustment with inverse probability of treatment weighting, baseline characteristics were well-balanced between the two groups, with the exception of transferrin saturation, hepcidin, total cholesterol, and vitamin B12 levels (Table 1; Supplementary Table S1). The spectrum of overt infections included pulmonary infections, peritoneal dialysis-related peritonitis, catheter-related infections, urinary tract infections, and other infection types. As summarized in Supplementary Table S2, the distribution of infection types did not differ significantly between the roxadustat and rHuEPO groups.

**TABLE 1:** Characteristics of patients in the roxadustat and rHuEPO groups (x ± s).

Parameters	Roxadustat (N = 88)	rHuEPO (N = 79)	*P* Value
Age (years)	56.8 ± 17.4	59.4 ± 14.6	0.662
Male, n (%)	58 (65.9)	47 (59.5)	0.451
BMI, kg/m^2^	22.9 ± 4.5	23.1 ± 3.8	0.710
Cause of kidney disease (%)			
Diabetes, n (%)	22 (25.0)	20 (25.3)	0.828
Hypertension, n (%)	19 (21.6)	18 (22.8)	0.613
Glomerulonephritis, n (%)	19 (21.6)	16 (20.3)	0.324
Other, n (%)	28 (31.8)	25 (31.6)	0.681
ACEI/ARB, n (%)	12 (13.6)	13 (16.5)	0.142
Type of dialysis			0.563
HD, n (%)	39 (44.3)	34 (43.2)	
PD, n (%)	49 (55.7)	45 (56.8)	
Duration of dialysis (months)	38.7 ± 14.4	39.3 ± 16.8	0.627
Residual renal function			
Anuria (<100 mL/d), n (%)	23 (26.1)	25 (31.6)	0.324
Oliguria (100 ∼ 400 mL/d), n (%)	27 (30.7)	22 (27.8)	0.512
Normal urine (>500 mL/d), n (%)	38 (43.2)	32 (40.5)	0.162
Type of vascular access in HD			
Venous catheter, (n,%)	8 (20.5)	10 (22.2)	0.353
AV fistula, (n,%)	31 (79.5)	35 (77.8)	0.242
SBP (mmHg)	140.7 ± 25.5	138.1 ± 26.4	0.678
DBP (mmHg)	87.0 ± 17.9	81.6 ± 16.1	0.352
Hemoglobin (g/L)	90.3 ± 15.2	91.9 ± 17.8	0.774
Reticulocyte (%)	2.2 ± 1.2	2.5 ± 1.9	0.562
Red blood cell volume distribution width (%)	14.6 ± 2.0	14.5 ± 1.9	0.735
TSAT (%)	28.7 (18.2, 35.8)	18.9 (14.3, 33.2)	**0.046**
Ferritin (ng/mL)	722.1 (433.9, 889.5)	688.3 (528.6, 797.1)	0.752
Hepcidin (ng/mL)	71.5 ± 10.6	97.6 ± 12.9	**0.032**
white blood (×10^9^)	9.16 ± 5.1	7.7 ± 2.9	0.056
Proportion of neutrophils (%)	89.5 ± 3.2	90.1 ± 4.3	0.783
CRP (mg/L)	32.4 (6.5, 74.8)	35.9 (7.7, 92.6)	0.186
≤ULN (n,%)	13 (14.8)	12 (15.2)	0.743
>ULN (n,%)	75 (85.2)	67 (84.8)	0.323
PCT (ng/mL)	0.96 (0.3, 3.1)	0.85 (0.4, 2.8)	0.236
≤ULN (n,%)	15 (17.0)	16 (20.3)	0.653
>ULN (n,%)	73 (83.0)	63 (79.7)	0.257
Type of infections			
Dialysis-related infections (peritonitis and HD catheter infection), (n,%)	33 (37.5)	26 (32.9)	0.219
Non-dialysis-related infections (pulmonary or urinary tract infections), (n,%)	64 (72.7)	58 (73.4)	0.318

^a^ Variables are presented as the mean ± SD, median (interquartile range) or n (%).

Abbreviations: BMI, body mass index; ACEI/ARB, angiotensin-converting enzyme inhibitor/angiotensin II, receptor blocker; ULN, upper limit of normal; CRP, C-reactive protein (ULN, 9.0 mg/L); PCT, procalcitonin (ULN, 0.5 ng/mL); HDL-C, high density lipoprotein, cholesterol; LDL-C, low density lipoprotein cholesterol; NDD, not receiving dialysis; HD, hemodialysis; PD, peritoneal dialysis; SBP, systolic blood pressure; DBP, diastolic blood pressure; TSAT, transferrin saturation; PTH, parathyroid hormone.

Bold values indicates a statistical difference of *P* < 0.05.

### Hemoglobin changes, iron metabolism and hepcidin levels during active infection

Three key time points were defined based on the clinical course of overt infection: time of infection onset (T1), time of symptomatic improvement (T2), and end of follow-up (T3). The interval from T1 to T2 was 10.6 ± 3.3 days (roxadustat group: 9.6 ± 2.7 days; rHuEPO group: 10.2 ± 4.3 days), and from T2 to T3 was 30.4 ± 7.6 days (roxadustat: 30.2 ± 5.6 days; rHuEPO: 30.7 ± 4.8 days). During the observation period (T1 to T3), the mean weekly dose was 98 ± 14 mg for roxadustat and 9150 ± 294 IU for rHuEPO.

**FIGURE 2:**
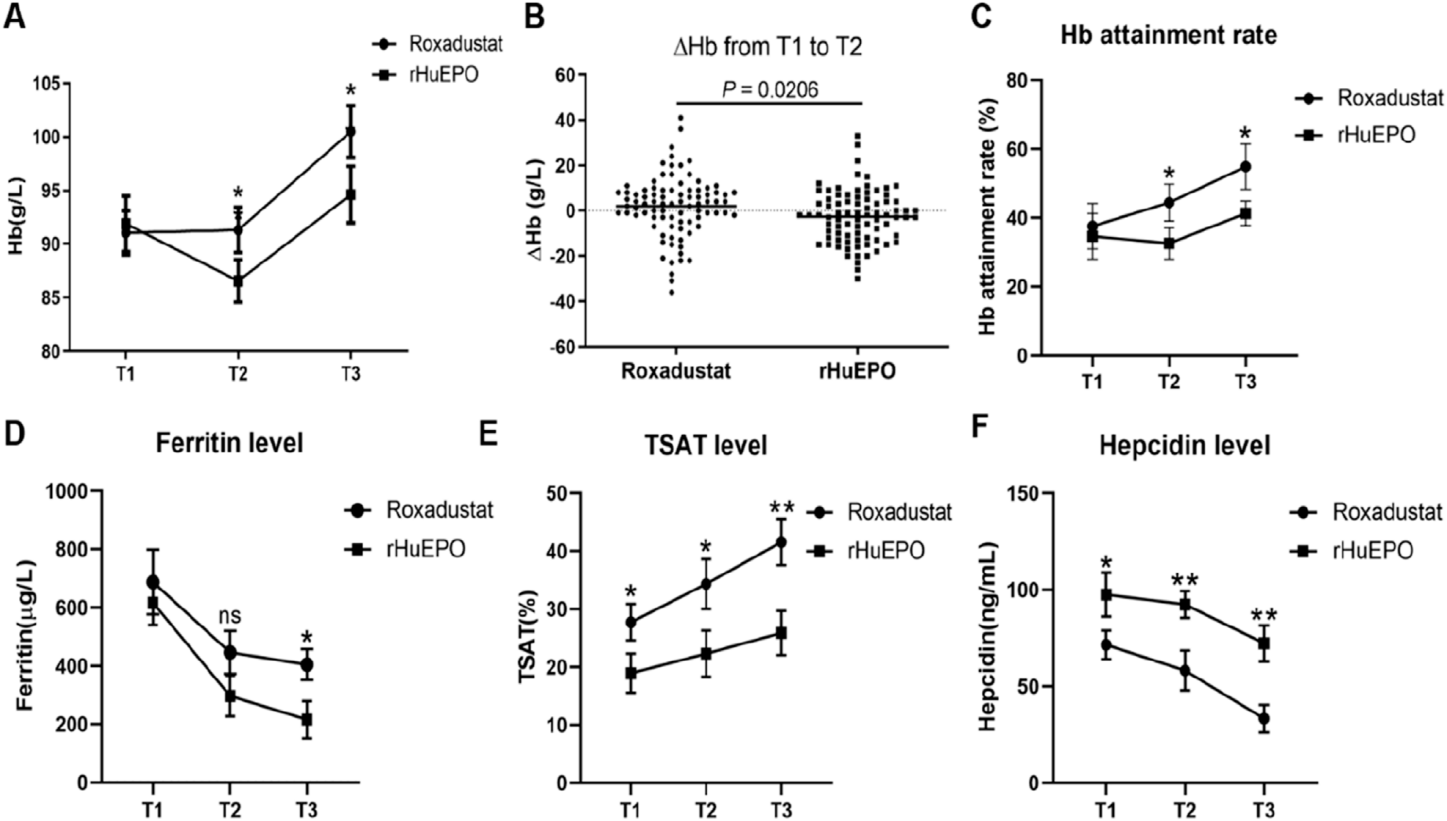
Changes in Hb and iron metabolism before and after infection. **(A)** Comparison of the Hb values in the roxadustat and rHuEPO groups at T1,T2, and T3. **(B)** ΔHb in the roxadustat and rHuEPO groups from T1 to T2. **(C)** Hb attainment rate, **(D)** Ferritin level, **(E)** TSAT level, **(F)** Hepcidin level in the roxadustat and rHuEPO groups at T1,T2, and T3. T1: roxadustat (n = 88) vs. rHuEPO (n = 79), T2: roxadustat (n = 88) vs. rHuEPO (n = 79), T3: roxadustat (n = 80) vs. rHuEPO (n = 74), Missing follow-up data was obtained because some patients did not receive outpatient review. *P < 0.05 when comparing roxadustat with rHuEPO at T1, T2, and T3. **(B)** △Hb value from T1 to T2, *P* < 0.05 when comparing roxadustat and rHuEPO.

Compared with rHuEPO, roxadustat led to a significantly greater increase in hemoglobin levels following infection. At T2, hemoglobin levels were 92 ± 3.2 g/L in the roxadustat group versus 86 ± 2.6 g/L in the rHuEPO group. This advantage was maintained at the T3 follow-up (mean: 1 month), with hemoglobin concentrations rising to 101 ± 4.4 g/L in the roxadustat group and 94 ± 4.8 g/L in the rHuEPO group (Figure 2A). Hemoglobin changes differed significantly between groups over the infection period (Figure 2B). The weekly rate of Hb change (ΔHb) from T1 to T2 was 1.92 ± 14.71 g/L/week with roxadustat compared to −2.62 ± 11.70 g/L/week with rHuEPO. To evaluate the Hb attainment rate of roxadustat and rHuEPO, we defined the target Hb level as ≥110 g/L and ≤130 g/L, in accordance with KDIGO guideline recommendations. At T1, there was no significant difference in Hb attainment rates between the roxadustat and rHuEPO groups. However, by T2, roxadustat demonstrated a significantly higher Hb attainment rate compared to rHuEPO. This improvement became even more pronounced at T3 (Figure 2C).

As ferritin is an acute-phase reactant during infection, both the rHuEPO and roxadustat groups exhibited elevated ferritin levels at T1. By T2, ferritin levels had decreased in both groups, with no significant difference observed between them. However, at T3, during the follow-up period after infection resolution, a significant difference in ferritin levels emerged between the roxadustat and rHuEPO groups (Figure 2D). These findings suggest that during active infection, ferritin may not accurately reflect iron stores in anemic patients and could mask underlying iron deficiency. In contrast, transferrin saturation (TSAT) levels differed significantly between the roxadustat and rHuEPO groups from T1 to T3, and this difference became more pronounced as the infection resolved (Figure 2E). This indicates that roxadustat’s effect on iron recycling and utilization remains effective irrespective of infection status.

Since roxadustat is known to regulate hepcidin production, we further measured hepcidin levels at all three time points to better elucidate its role in managing renal anemia during infection. We found that during active infection (T1), hepcidin levels were elevated in both groups, but were significantly higher in the roxadustat group compared to the rHuEPO group. As the infection improved with treatment, hepcidin levels declined in both groups, yet remained significantly different between the two groups at T2 and T3 (Figure 2F).

### Influencing factors of clinical efficacy of roxadustat among dialysis patients with active infection

To further explore potential confounding factors affecting the efficacy of roxadustat or rHuEPO during active infection, we performed a bivariate correlation analysis to evaluate the associations between multiple clinical variables and changes in hemoglobin (Hb) levels. The analysis included ΔHb as the dependent variable and the following independent variables: treatment type (roxadustat or rHuEPO), gender, age, BMI, dialysis vintage, use of ACEIs or ARBs, cause of kidney disease, dialysis modality, residual renal function, presence of arteriovenous fistula, type of vascular access (in HD patients), and changes in systolic (SBP) and diastolic blood pressure (DBP). As summarized in Supplementary Table S3, several factors were significantly correlated with ΔHb, including treatment assignment, TSAT, hepcidin, dialysis modality, residual renal function, type of infection, and PCT levels. Subsequent multiple linear regression analysis (Supplementary Table S4) confirmed that improvements in TSAT, hepcidin, dialysis modality, residual renal function, infection type, and PCT were independently associated with greater increases in ΔHb, indicating a correlation with amelioration of renal anemia.

To further elucidate the advantage of roxadustat in managing renal anemia during active infection, univariate and multivariate analyses were performed incorporating the previously identified independent factors and hemoglobin changes observed in the roxadustat group. The analysis demonstrated that TSAT, hepcidin, dialysis modality, residual renal function, infection type, and PCT levels play critical roles in mediating the efficacy of roxadustat under active infection conditions (Table 2).

**TABLE 2:** Univariate and multivariate analyses of influencing factors for clinical efficacy of roxadustat during infection.

	Univariate analyses			Multivariate analyses		
Parameters	*β*	OR (95% Cl)	*P*	*β*	OR (95% Cl)	*P*
TSAT (%)	0.038	1.386 (1.012, 1.762)	0.025*	0.056	1.266 (1.013, 1.521)	0.031*
Hepcidin (ng/mL)	0.019	1.211 (0.867, 1.555)	0.033*	0.015	1.197 (1.101, 1.293)	0.012*
Type of dialysis	−0.021	0.987 (0.843, 1.133)	0.013*	−0.033	0.961 (0.904, 1.018)	0.008**
Type of infections	0.018	1.023 (0.879, 1.167)	0.042*	0.002	1.011 (1.001, 1.021)	0.018*
PCT (ng/mL)	0.132	1.327 (0.895, 1.761)	0.027*	0.121	1.283 (0.998, 1.568)	0.006**
Residual renal function	0.049	1.125 (0.991, 1.259)	0.011*	0.037	1.108 (1.010, 1.206)	0.021*
Total cholesterol (mmol/L)	0.002	1.037 (0.975, 1.099)	0.483			
Vit B12 (pg/mL)	0.056	1.006 (0.827, 1.185)	0.120			

95% CI, 95% confidence interval. * indicates statistical difference *P* < 0.05, ** indicates statistical difference *P* < 0.01.

### Impact of dialysis modality on anemia treatment outcomes

**FIGURE 3:**
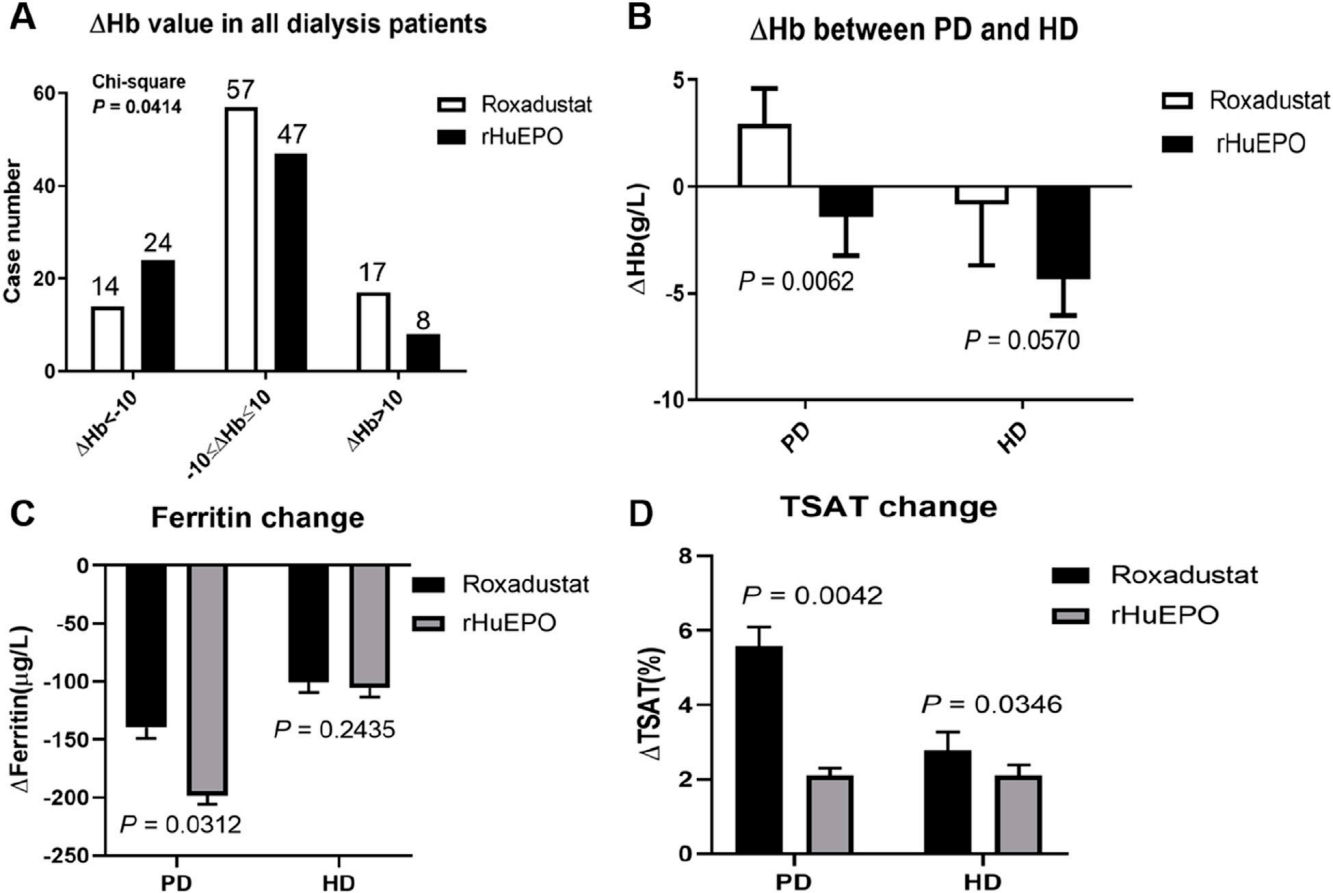
Hb levels in patients treated via different dialysis modalities before and after infection. **(A)** The number of all dialysis patients based on △Hb. **(B)** △Hb values of PD patients and HD patients treated with roxadustat (PD, n = 49; HD, n = 39) and rHuEPO (PD, n = 34; HD, n = 45). **(C)** Ferritin change of PD patients and HD patients treated with roxadustat or rHuEPO from T1 to T2. **(D)** TSAT change of PD patients and HD patients treated with roxadustat or rHuEPO from T1 to T2.

Patients exhibiting any variation in hemoglobin (Hb) levels were categorized as △Hb < −10 g/L, −10 g/L≤△Hb ≤ 10 g/L, or △Hb > 10 g/L (Figure 3A) among the entire cohort of dialysis patients. A significant difference in the change of hemoglobin (△Hb) was observed between the roxadustat group and the rHuEPO group from T1 to T2 (*P* = 0.0414). Among dialysis-dependent patients, anti-infective treatment led to varying degrees of alteration in hemoglobin levels. Specifically, PD patients exhibited a significantly greater △Hb in the roxadustat group compared to the rHuEPO group. In contrast, HD patients showed a reduction in △Hb in both treatment groups, with no statistically significant difference between roxadustat and rHuEPO (Figure 3B).

Further analysis of iron metabolism parameters revealed distinct differences between PD and HD patients treated with either roxadustat or rHuEPO for anemia of renal disease during active infection. During the period from T1 to T2, PD patients receiving rHuEPO exhibited a more pronounced decline in ferritin levels compared to those receiving roxadustat. In contrast, no significant effect on ferritin was observed in HD patients treated with either agent (Figure 3C). Regardless of dialysis modality, roxadustat resulted in a greater increase in transferrin saturation (TSAT) compared to rHuEPO (Figure 3D).

### Relationship between Hb and overt infections

**FIGURE 4:**
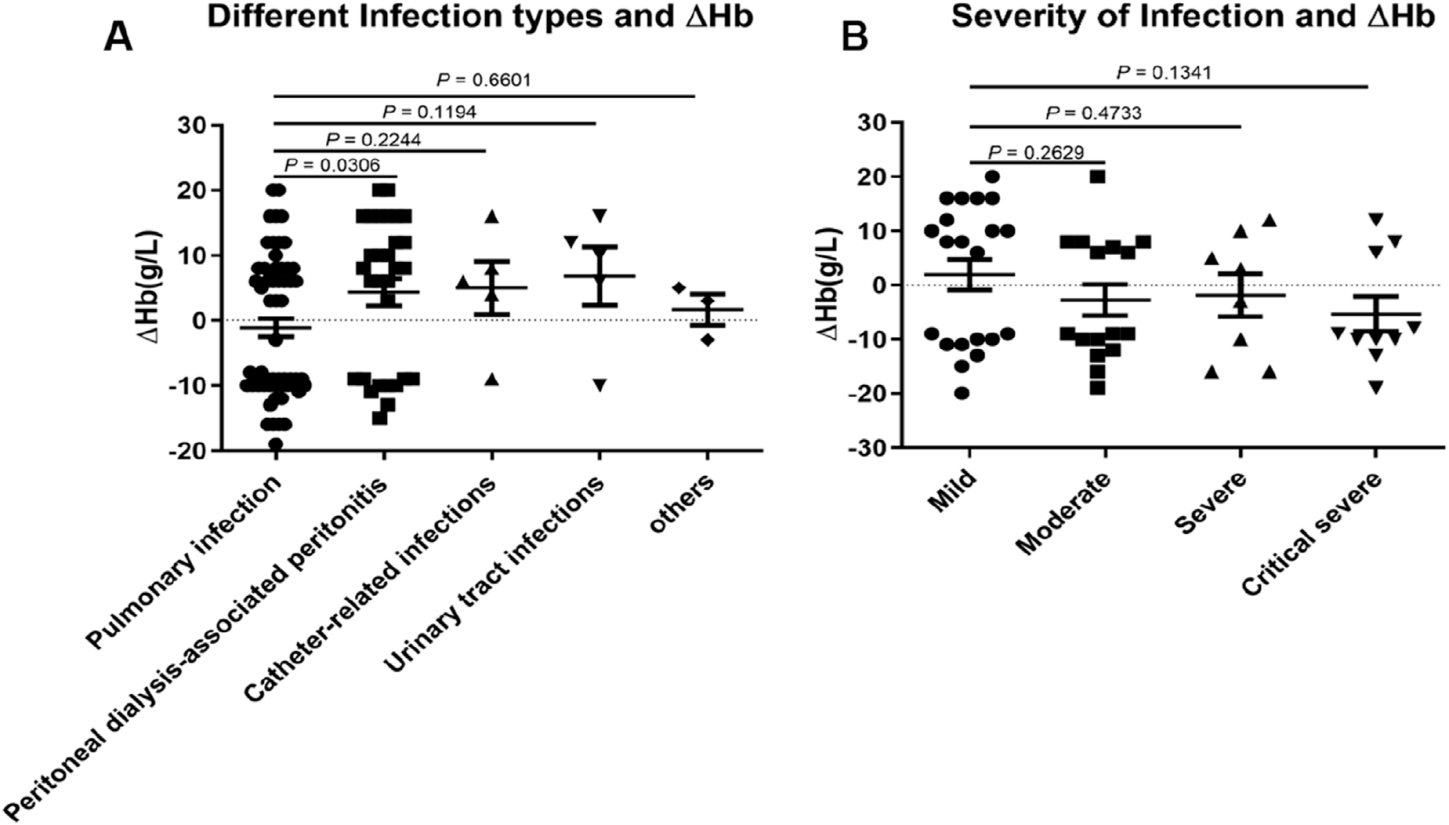
The effect of infection type and infection severity on △Hb in dialysis patients administered roxadustat. **(A)** Different infection types and △Hb levels in patients with pulmonary infection (n = 40), peritoneal dialysis-associated peritonitis (n = 24), catheter-related infections (n = 5), urinary tract infections (n = 5), and other infections (n = 3). **(B)** The effect of infection severity on △Hb based on PCT >ULN (n = 63), mild (n = 29), moderate (n = 16), severe (n = 8), and critical severity (n = 10).

To further elucidate factors influencing the efficacy of roxadustat in treating renal anemia among dialysis patients with overt infection, we assessed the impact of infection type and severity. Infection severity was stratified by procalcitonin (PCT) levels as follows: mild (<0.5), moderate (0.5∼2), severe (2∼10), and critical (≥10), as illustrated in Figure 4. Given its clinical significance, pulmonary infection was designated as the reference group.

The ΔHb value in patients with peritoneal dialysis-associated peritonitis differed significantly from that in the pulmonary infection group. Although no statistically significant differences were observed in other infection types, a trend toward higher ΔHb was noted in non-pulmonary infections compared to pulmonary infections (Figure 4A). No correlation was found between PCT levels and ΔHb values (data not shown).

When assessing infection severity based on PCT categories, no significant differences in ΔHb were detected between the mild group and other severity groups. However, more severe infections were associated with progressively lower ΔHb values (Figure 4B).

### Safety evaluation

Both the roxadustat and rHuEPO groups exhibited stable mean systolic blood pressure levels from T1 to T3, with no significant differences observed between the two groups (Data not shown). Similarly, mean diastolic blood pressure levels did not differ significantly between groups throughout the observation period. Mean serum potassium levels also remained stable from T1 to T3 in both groups. At T2, the mean serum potassium level was 4.1 ± 0.4 mmol/L in the roxadustat group and 4.3 ± 0.5 mmol/L in the rHuEPO group, with no statistically significant treatment difference between groups (*P* > 0.05). Comparable results were observed at T3.

## Discussion

Anemia in patients with CKD arises from multiple etiological factors. Among these, inflammation—frequently present as a comorbidity in renal anemia—represents a major contributor to treatment resistance (Takahashi et al., 2021). Inflammatory responses may stem from infectious or non-infectious causes, including confirmed pathogens, autoimmune disorders, and malignancies, often resulting in a persistent microinflammatory state. Recently, roxadustat has emerged as a potential therapeutic option for renal anemia accompanied by microinflammation (Akizawa et al., 2020; Fishbane et al., 2021; Fishbane et al., 2022; Barratt et al., 2021a). Nevertheless, its efficacy in dialysis patients with overt infection remains inadequately established. In this retrospective cohort study, we demonstrate that roxadustat effectively ameliorates anemia in dialysis patients suffering from overt infection.

Infection and microinflammation are known contributors to hyporesponsiveness to erythropoiesis-stimulating agents (ESAs) in patients with renal anemia (Weir, 2021). During active infection (T1 to T2), treatment with rHuEPO was associated with a decline in hemoglobin levels, consistent with ESA hyporesponsiveness. Although the present study indicated that overt infection did not significantly impair the efficacy of roxadustat, a reduction in hemoglobin was still observed in infected patients receiving Roxadustat. However, this decrease was less pronounced than in the rHuEPO group. The attenuated decline may be attributed to the unresolved infection during the observation period, as well as to the ability of roxadustat to modulate the HIF pathway, thereby reducing hepcidin levels and improving iron metabolism (Locatelli et al., 2017; Chen et al., 2017). These findings align with our data (Figure 3).

Iron deficiency represents a major cause of ESA hyporesponsiveness (Kidney Disease: Improving Global Outcomes KDIGO Anemia Work Group, 2012). Roxadustat may confer benefits in infected patients who are refractory to ESAs, primarily through enhancement of iron absorption and mobilization rather than solely through elevation of erythropoietin levels. After adjusting for confounding factors in dialysis patients through univariate and multivariate analyses (Wu et al., 2024; Fernández-Gallego et al., 2000; Wang et al., 2024; Xu et al., 2024), we found that improvements in several parameters—including TSAT, hepcidin levels, dialysis modality, residual renal function, infection type, and PCT levels—were associated with a more pronounced efficacy of roxadustat compared to rHuEPO in infected dialysis patients. Thus, HIF-PH inhibitors such as roxadustat represent a promising therapeutic alternative for CKD patients with anemia and concurrent infection, particularly those exhibiting hyporesponsiveness to conventional ESA therapy.

The prevalence of CKD progressing to end-stage kidney disease (ESKD) is increasing, leading to a growing population of patients requiring PD or HD (He et al., 2020). PD offers several advantages over HD in preserving residual renal function (RRF), which in turn exerts beneficial effects on mitigating inflammation and improving anemia (Tanriover et al., 2022). In the present study, roxadustat produced a greater increase in ΔHb than rHuEPO during the infection period (T1 to T2) among PD patients, despite no additional dosing of either agent during infection treatment. Although no significant difference in ΔHb was observed between roxadustat and rHuEPO in HD patients with overt infection, the ΔHb in the roxadustat group was numerically lower. Furthermore, our results suggest that roxadustat may enhance iron utilization potentially through regulation of iron metabolism, with PD patients appearing to benefit more markedly from roxadustat therapy. This effect may be attributable to promoted erythropoietin production, improved iron metabolism, and reduced hepcidin levels associated with overt infection in PD patients who retain RRF. These results are consistent with findings demonstrating the superiority of roxadustat over rHuEPO in non-infected peritoneal dialysis patients (Li et al., 2024; Gan et al., 2020) and its significant efficacy in improving anemia among CKD patients with systemic microinflammation (Tu et al., 2024). Collectively, these findings indicate that roxadustat may be more effective than rHuEPO in improving renal anemia among PD patients with overt infection.

In the pivotal global trials of roxadustat, a pooled analysis of dialysis-dependent chronic kidney disease (DD-CKD) patients demonstrated that the incidence of severe and fatal infections was comparable between the roxadustat and epoetin alfa groups (Barratt et al., 2021b). Accordingly, conventional doses of either roxadustat or rHuEPO were administered in this study to dialysis patients with renal anemia and overt infection, alongside appropriate antibacterial therapy. Our results indicate that roxadustat use in infected patients was associated with reduced hemoglobin fluctuations and more effective hemoglobin correction during the short-term period from T1 to T3, facilitating more rapid anemia improvement and potentially mitigating adverse effects related to hemoglobin variability and sustained low hemoglobin levels.

While phase III clinical studies utilized CRP levels to assess the effect of roxadustat in dialysis patients with microinflammation (Chen et al., 2019), the present study employed PCT as a biomarker for overt infection (particularly bacterial infection). Although there was no statistically significant difference in the distribution of ΔHb when comparing within different infection types or within different levels of infection severity, the correlation analyses revealed a significant association between ΔHb and infection type or severity. Our findings suggest that the efficacy of roxadustat still be influenced by these factors in dialysis patients with renal anemia and overt infection.

Although this single-center retrospective study has a limited sample size and does not fully elucidate the mechanisms by which infection-induced inflammation exacerbates renal anemia, it provides the first clinical demonstration that roxadustat significantly improves anemia and iron metabolism in dialysis patients with overt infection.

## Data Availability

The original contributions presented in the study are included in the article/Supplementary Material, further inquiries can be directed to the corresponding authors.
