# Supplementary material for: Impact of roxadustat on anemia management in infected patients undergoing long-term dialysis: a retrospective cohort analysis
Source: Front Pharmacol. 2025 Oct 8;16:1695376. doi: 10.3389/fphar.2025.1695376 (PMC12540434; doi:10.3389/fphar.2025.1695376)
Supplement: Supplementary file 1 [file Table1.docx]

**Supplementary material**

**Methods**

The exclusion criteria were as follows: (1) bleeding, (2) transfusions in the first 3 months and during the follow-up period, (3) malignant tumors or autoimmune diseases, (4) cross-use of roxadustat and rHuEPO, (5) Non-bacterial infections (Excluding fungal and viral infections), and (6) incomplete anemia-related data. (7) Patients who demonstrated no improvement after 15 days of antibiotic therapy or who died during the course of treatment were excluded.

**Table S1**. Laboratory baseline parameters at T1.

| Parameters | Roxadustat  **(N= 88)** | rHuEPO  **(N= 79)** | ***P* value** |
| --- | --- | --- | --- |
| Total cholesterol (mmol/L) | 3.8 ± 1.5 | 4.5 ± 2.9 | **0.036** |
| Triglyceride (mmol/L) | 1.8 ± 0.8 | 1.5 ± 0.9 | 0.561 |
| HDL-C (mmol/L) | 0.8 ± 0.3 | 1.0 ± 0.5 | 0.651 |
| LDL-C (mmol/L) | 2.0 ± 0.9 | 2.18 ± 1.2 | 0.868 |
| Folic acid (ng/mL) | 19.3 ± 12.6 | 16.3 ± 12.4 | 0.383 |
| Vit B12 (pg/mL) | 657.3 ± 294.0 | 874.2 ± 522.0 | **0.035** |
| PTH (pg/mL) | 386.4 ± 463.8 | 410.1 ± 452.6 | 0.326 |
| Serum phosphate (mmol/L) | 1.8 ± 0.3 | 1.9 ± 0.5 | 0.213 |
| K^+^ (mmol/L) | 4.2 ± 0.2 | 4.5 ± 0.3 | 0.483 |

**Information about overt infections in dialysis patients with renal anemia**

A total of 167 dialysis patients with renal anemia and overt infection who were treated with either roxadustat or rHuEPO were enrolled. The spectrum of overt infections included pulmonary infections, peritoneal dialysis-associated peritonitis, catheter-related infections, urinary tract infections, and other infection types. Among the cohort, pulmonary infections were the most common (n = 104, 62.3%), followed by peritoneal dialysis-associated peritonitis (n = 49, 28.0%), catheter-related infections (n = 11, 6.0%), urinary tract infections (n = 9, 5.0%), and other infections-including cyst infection in polycystic kidney disease (n = 9, 5.0%). The majority of patients (n = 148, 88.6%) presented with a single infection site, while 19 (11.4%) had dual infections. As summarized in Table 2, the distribution of infection types did not differ significantly between the roxadustat and rHuEPO groups.

**Table S2.** The types of overt infections in dialysis patients with renal anemia

| Types of overt infections | Roxadustat  **(N= 88)** | rHuEPO  **(N= 79)** |
| --- | --- | --- |
| Pulmonary infection (n,%) | 56 (63.6) | 48 (60.8) |
| Peritoneal dialysis-associated peritonitis (n,%) | 29 (33.0) | 20 (25.3) |
| Catheter-related infections (n,%) | 5 (5.7) | 6 (7.6) |
| Urinary tract infections(n,%) | 5 (5.7) | 4 (5.1) |
| Other (n,%) | 3 (3.4) | 6 (7.6) |
| Single infection (n,%) | 77 (87.5) | 71 (89.9) |
| Double infection (n,%) | 11 (12.5) | 8 (10.1) |

*Variables are presented as the mean ± SD, median (interquartile range) or n (%).

**Table S3**. Relationships between changes in Hb and clinical parameters

| Parameters | **Correlation coefficient** | ***P*** |
| --- | --- | --- |
| Treatment(Roxadustat or rHuEPO) | 0.325 | **0.026** |
| Age (years) | -0.078 | 0.267 |
| Male, n (%) | 0.049 | 0.312 |
| BMI, kg/m^2^ | 0.012 | 0.429 |
| Cause of kidney disease (%) | 0.028 | 0.721 |
| ACEI/ARB, n (%) | -0.067 | 0.218 |
| Type of dialysis | 0.412 | **0.019** |
| Duration of dialysis (months) | -0.079 | 0.127 |
| Residual renal function | 0.387 | **0.039** |
| Type of vascular access in HD | 0.081 | 0.739 |
| SBP (mmHg) | 0.091 | 0.832 |
| DBP (mmHg) | 0.082 | 0.739 |
| Reticulocyte (%) | -0.092 | 0.349 |
| Red blood cell volume distribution width (%) | -0.087 | 0.518 |
| TSAT (%) | 0.453 | **0.017** |
| Ferritin (ng/mL) | 0.092 | 0.083 |
| Hepcidin (ng/mL) | 0.393 | **0.029** |
| white blood (×10^9^) | -0.056 | 0.822 |
| Proportion of neutrophils (%) | -0.067 | 0.667 |
| CRP (mg/L) | 0.217 | 0.068 |
| PCT (ng/mL) | 0.387 | **0.036** |
| Total cholesterol (mmol/L) | 0.039 | 0.839 |
| Triglyceride (mmol/L) | 0.002 | 0.218 |
| HDL-C (mmol/L) | 0.011 | 0.313 |
| LDL-C (mmol/L) | 0.028 | 0.673 |
| Folic acid (ng/mL) | -0.033 | 0.548 |
| Vit B12 (pg/mL) | 0.209 | 0.073 |
| PTH (pg/mL) | -0.018 | 0.912 |
| Serum phosphate (mmol/L) | 0.102 | 0.756 |
| K^+^ (mmol/L) | -0.132 | 0.194 |
| Type of infections | -0.323 | **0.018** |

Table S4.Relationships between changes in Hb and clinical parameters in the dialysis population with infection according to multiple linear regression analysis

| Parameters | **Regression coefficients** | **95.0% CI** | | ***P*** |
| --- | --- | --- | --- | --- |
| (Constant) | 21.394 | -32.198 | 74.782 | 0.518 |
| Treatment (Roxadustat or rHuEPO) | 0.596 | -0.219 | 1.186 | **0.021** |
| Age (years) | -0.102 | -0.421 | 0.283 | 0.297 |
| Male, n (%) | 0.328 | -0.065 | 0.673 | 0.468 |
| BMI, kg/m^2^ | 0.008 | -0.398 | 0.386 | 0.128 |
| Cause of kidney disease (%) | 0.049 | -0.358 | 0.401 | 0.020 |
| ACEI/ARB, n (%) | -0.364 | -0.728 | 0.169 | 0.329 |
| Type of dialysis | 0.398 | -0.112 | 0.769 | **0.012** |
| Duration of dialysis (months) | -0.263 | -0.629 | 0.302 | 0.379 |
| Residual renal function | 0.161 | -0.428 | 0.677 | **0.028** |
| Type of vascular access in HD | -1.202 | -4.231 | 2.389 | 0.632 |
| △SBP (mmHg) | 0. 912 | -1.286 | 3.282 | 0.283 |
| △DBP (mmHg) | -1.218 | -5.212 | 3.868 | 0.312 |
| △Reticulocyte (%) | -0.273 | -2.143 | 2.389 | 0.191 |
| △Red blood cell volume distribution width (%) | -0.189 | -1.822 | 2.126 | 0.523 |
| △TSAT (%) | 0.318 | -2.219 | 3.126 | **0.019** |
| △Ferritin (ng/mL) | -0.129 | -0.928 | 0.732 | 0.126 |
| △Hepcidin (ng/mL) | -0.126 | -0.323 | 0.106 | **0.031** |
| △white blood (×10^9^) | -0.249 | -0.635 | 0.286 | 0.783 |
| △Proportion of neutrophils (%) | 0.127 | -0.398 | 0.655 | 0.698 |
| △CRP (mg/L) | 0.519 | -1.236 | 2.312 | 0.102 |
| △PCT (ng/mL) | 0.193 | -2.128 | 2.653 | **0.008** |
| △Total cholesterol (mmol/L) | -0.124 | -0.632 | 0.488 | 0.923 |
| △Triglyceride (mmol/L) | 0.125 | -0.611 | 0.817 | 0.233 |
| △HDL-C (mmol/L) | 0.329 | -0.298 | 1.026 | 0.702 |
| △LDL-C (mmol/L) | 0.238 | -0.631 | 1.125 | 0.127 |
| △Folic acid (ng/mL) | 0.214 | -0.129 | 0.566 | 0.387 |
| △Vit B12 (pg/mL) | 0.322 | -0.627 | 1.022 | 0.065 |
| △PTH (pg/mL) | -0.289 | -0.797 | 0.376 | 0.421 |
| △Serum phosphate (mmol/L) | 0.294 | -0.612 | 1.129 | 0.632 |
| △K^+^ (mmol/L) | -0.357 | -2.821 | 2.366 | 0.169 |
| Type of infections | -0.653 | -3.212 | 4.102 | **0.041** |
